# Accelerated estimation and permutation inference for ACE modeling

**DOI:** 10.1002/hbm.24611

**Published:** 2019-04-29

**Authors:** Xu Chen, Elia Formisano, Gabriëlla A. M. Blokland, Lachlan T. Strike, Katie L. McMahon, Greig I. de Zubicaray, Paul M. Thompson, Margaret J. Wright, Anderson M. Winkler, Tian Ge, Thomas E. Nichols

**Affiliations:** ^1^ Department of Statistics University of Warwick Coventry UK; ^2^ Department of Cognitive Neuroscience, Faculty of Psychology and Neuroscience Maastricht University Maastricht the Netherlands; ^3^ Maastricht Centre for Systems Biology (MaCSBio) Maastricht University Maastricht the Netherlands; ^4^ Department of Biomedical Data Sciences Leiden University Medical Center Leiden the Netherlands; ^5^ Psychiatric and Neurodevelopmental Genetics Unit, Center for Genomic Medicine Massachusetts General Hospital Boston Massachusetts; ^6^ Stanley Center for Psychiatric Research Broad Institute of MIT and Harvard Cambridge Massachusetts; ^7^ Department of Psychiatry Massachusetts General Hospital, Harvard Medical School Boston Massachusetts; ^8^ Queensland Brain Institute University of Queensland Brisbane Queensland Australia; ^9^ Centre for Advanced Imaging University of Queensland Brisbane Queensland Australia; ^10^ Faculty of Health and Institute of Health and Biomedical Innovation Queensland University of Technology Brisbane Queensland Australia; ^11^ Imaging Genetics Center University of Southern California Los Angeles California; ^12^ Emotion and Development Branch National Institute of Mental Health, National Institutes of Health Bethesda Maryland; ^13^ Department of Psychiatry Yale University School of Medicine New Haven Connecticut; ^14^ Athinoula A. Martinos Center for Biomedical Imaging Massachusetts General Hospital Charlestown Massachusetts; ^15^ Oxford Big Data Institute, Li Ka Shing Centre for Health Information and Discovery, Nuffield Department of Population Health University of Oxford Oxford UK

**Keywords:** ACE model, heritability inference, permutation test, twin studies

## Abstract

There are a wealth of tools for fitting linear models at each location in the brain in neuroimaging analysis, and a wealth of genetic tools for estimating heritability for a small number of phenotypes. But there remains a need for computationally efficient neuroimaging genetic tools that can conduct analyses at the brain‐wide scale. Here we present a simple method for heritability estimation on twins that replaces a variance component model‐which requires iterative optimisation‐with a (noniterative) linear regression model, by transforming data to squared twin‐pair differences. We demonstrate that the method has comparable bias, mean squared error, false positive risk, and power to best practice maximum‐likelihood‐based methods, while requiring a small fraction of the computation time. Combined with permutation, we call this approach “Accelerated Permutation Inference for the ACE Model (APACE)” where ACE refers to the additive genetic (A) effects, and common (C), and unique (E) environmental influences on the trait. We show how the use of spatial statistics like cluster size can dramatically improve power, and illustrate the method on a heritability analysis of an fMRI working memory dataset.

## 1 INTRODUCTION

There continues to be growing interest in the joint study of imaging phenotypes and genetic data (genotypes; Glahn, Thompson, & Blangero, 2007). Imaging genetics is a multidisciplinary research area investigating the genetic influences on brain structure and function using both imaging and genetic information. A phenotype is an observable characteristic that results from the interaction of genetic inheritance and environmental conditions. To quantify the degree of the genetic effects on a phenotype, heritability is defined as the proportion of phenotypic variation that is due to genetic factors, where the genetic variability can be attributed to a particular gene or the aggregate of multiple genes. Several studies have examined the heritability of psychiatric disorders, and many of them suggest that most psychiatric disorders are moderately to highly heritable, with an estimated heritability of 0.83 for schizophrenia (Cannon, Kaprio, Lönnqvist, Huttunen, & Koskenvuo, 1998) and 0.85 for bipolar affective disorder (McGuffin et al., 2003). There also exists a large number of neuroimaging studies investigating the heritability of neuroanatomical phenotypes and brain functions and reporting considerable heritability (see for example, Ge et al., 2015, 2016; Ge, Holmes, Buckner, Smoller, & Sabuncu, 2017; Glahn et al., 2010; Thompson, Ge, Glahn, Jahanshad, & Nichols, 2013).

Recently, the development of genomic technologies has allowed direct heritability analysis from unrelated individuals using genome‐wide genetic data (Ge, Chen, Neale, Sabuncu, & Smoller, 2017; Yang et al., 2010; Yang, Lee, Goddard, & Visscher, 2011). Without genetic data, heritability can be estimated by studying individuals with varying degrees of genetic relatedness. Classic twin studies are often employed to estimate the level of genetic and environmental variations in traits. The method of moments and the maximum likelihood approach are most commonly used methods to estimating heritability. Falconer's formula provides a simple point estimator for heritability based on moment matching (Falconer & Mackay, 1996). The best practice, likelihood‐based method uses the variance component model, which parameterizes different degrees of covariance expected with varying relatedness between subjects; the variance parameters are estimated by applying the maximum likelihood criterion (Neale & Cardon, 1992).

While the first neuroimaging studies measuring heritability used Falconer's method (e.g., Wright, Sham, Murray, Weinberger, & Bullmore, 2002), the likelihood‐based approach is now routine, with variance components or structural equation modeling (SEM) methods applied one voxel at a time. However, such methods cannot exploit the spatial nature of the data, nor can they provide accurate inferences corrected for family‐wise error rate over the brain. Although a simple Bonferroni correction offers the control of family‐wise error rate, it is typically quite conservative for smooth images. When feasible, permutation inference offers an exact control of false positive risk and allows for specialized spatial statistics, such as inference by cluster size, which delivers family‐wise error rate corrections while implicitly accounting for spatial dependence. However, most commonly used software tools for heritability estimation using twin data at present are too slow and unreliable to allow permutation.

In this article, we propose a linear regression‐based method that is new to the neuroimaging community, based on the method of Grimes and Harvey (1980) and closely related to the Haseman–Elston regression for genetic linkage studies (Ge et al., 2018; Haseman & Elston, 1972). It allows voxel‐wise heritability estimation with an approximate but remarkably fast and accurate performance. Using detailed Monte Carlo evaluations, we demonstrate that this method is valid with controlled false positive risk, and its statistical power is comparable to existing methods. With the speed advantage, this newly proposed method makes permutation inference more feasible and applicable. Here we also present for the first time our permutation approach in detail, which is developed for both voxel‐ and cluster‐wise inferences, with an application to a real fMRI blood oxygen level dependent (BOLD) dataset. Aside from fMRI data, this approach can also be applied to any other type of neuroimaging data.

## 2 THEORY AND METHODS

Heritability can be interpreted as the proportion of phenotypic variance explained by a single genetic marker (Filippini et al., 2008) or any subset of genes/markers of the genome (Stein et al., 2010). To quantify heritability, the total phenotypic variance ($\sigma_{P}^{2}$) can be partitioned into genetic ($\sigma_{G}^{2}$) and environmental ($\sigma_{E}^{2}$) components (Falconer & Mackay, 1996) in a linear manner:

$$\sigma_{P}^{2}=\sigma_{G}^{2}+\sigma_{E}^{2}.$$

The heritability in the broad sense (*H* ^2^) measures the overall genetic influence on a trait, defined by

$$H^{2}=\frac{\sigma_{G}^{2}}{\sigma_{P}^{2}},$$

where the genetic variation $\sigma_{G}^{2}$ summarizes the additive and nonadditive genetic contributions. The additive genetic effect arises from the linear addition of independent genes, or more technically, allelic contributions at different gene loci, while the nonadditive genetic effect refers to, for example, dominance or the interactive influence among alleles within or between gene loci (e.g., epistasis). The additive genetic variation is generally of more interest since it is the summed effects of a particular allele or alleles at a given locus or at multiple trait‐related loci. Thus, the narrow‐sense heritability is defined as the proportion of phenotypic variation accounted for by the additive genetic effect ($\sigma_{A}^{2}$) with an expression of

$$h^{2}=\frac{\sigma_{A}^{2}}{\sigma_{P}^{2}},$$

which is more commonly used and is usually just called “heritability”. We now detail the models employed to assess heritability using twin data.

### 2.1 The model

#### 2.1.1 Twin studies and ACE modeling

Normally twins are categorized as identical or monozygotic (MZ) and fraternal or dizygotic (DZ) twins. MZ twins have identical genotypes and DZ twins share, on average, 50% of their gene variants, which leads to the assumption of differential levels of sharing of additive genetic effects. Even in the absence of genetic influences on a phenotype, it is likely that twins are phenotypically more similar than unrelated individuals since they have been raised in the same family environment. This gives rise to the common environmental factor, which contributes to the covariance within twin pairs regardless of MZ or DZ type. Finally, there is an independent unique error, corresponding to the usual independent and identically distributed (i.i.d.) noise corrupting the measurements plus actual unique environmental influences, for example, trauma and illness. The phenotypic variance ($\sigma_{P}^{2}$) within the population is assumed to be the same and can be divided into additive genetic (*A*), common environmental (*C*), and unique environmental (*E*) components, written as

$$\sigma_{P}^{2}=A+C+E.$$

The so‐called ACE modeling in twin studies is based on this variance decomposition (Lee et al., 2010). Narrow‐sense heritability is denoted by

(1) $$h^{2}=\frac{A}{A+C+E},$$

and similarly, the contribution of common environmental factor can be defined as

(2) $$c^{2}=\frac{C}{A+C+E},$$

which describes the relative variance attributable to common environmental causes. The estimation of heritability and common environmental variance constitutes the analysis of variance components.

#### 2.1.2 Notation

In this section, we will outline the notation used in this article. Assume the experiment consists of *n* participants, including *n*_MZ_ MZ twins (*n*_MZ_/2 pairs), *n*_DZ_ DZ twins (*n*_DZ_/2 pairs), and *n*_S_ singletons (unrelated subjects and denoted by S), such that *n* = *n*_MZ_ + *n*_DZ_ + *n*_S_. For each (brain) image, with *V* voxels per subject, Y_*i*, *v*_ denotes the data from subject *i* and voxel *v* (*v* = 1, …, *V*). For voxel *v*, the data from all subjects can be written as a column vector **Y**_*v*_.

Some types of brain imaging data are directly measured, for example, grey matter density, producing one image per subject. However, fMRI requires hundreds of scans per subject to estimate blood flow change. A within‐subject model is often fitted to the imaging data for each subject, producing an image of BOLD effect magnitude for each subject (Frackowiak et al., 2004). Since the same form of model is fit at each voxel, going forward we suppress the voxel index *v*. Thus, the general linear model (GLM) in a matrix form for each voxel can be constructed as

(3) Y=Xβ+ɛ,

where **X** is an *n* × *p* design matrix including *p* − 1 covariates, and *ɛ* is the error vector, assumed to be normally distributed, written as **ɛ**~ℕ(**0**, **V**); the covariance matrix **V** is defined below. Typical covariates include age, sex, or other between‐subject effects.

To simplify the description of variance/covariance decomposition, we introduce a subject type indicator function T: {1,  … , *n*} → {MZ, DZ, S}. The function $T\left(\cdot \right)$ associates subject index *i* to subject type: $T\left(i\right)\in \left\{\mathrm{MZ},\mathrm{DZ},S\right\}\;\text{for}\;i=1,\ldots ,n$; that is, $T\left(i\right)=\mathrm{MZ}$ when subject *i* is an MZ twin, $T\left(i\right)=\mathrm{DZ}$ when subject *i* is a DZ twin, and $T\left(i\right)=S$ when subject *i* is a singleton. We now consider different possible covariance structures for pairs of subjects (*i*, *j*). To identify twins, let *j*(*i*) be the index of the twin pair of subject *i*. The MZ twin covariance can then be written, for *i* with $T\left(i\right)=T\left(j\left(i\right)\right)=\mathrm{MZ}$, as

(4) $$\mathbb{C}\text{ovMZ}=\mathrm{\mathbb{C}ov}\left(\begin{array}{c} Y_{i}\\ Y_{j\left(i\right)} \end{array}\right)=\left(\begin{array}{cc} A+C+E & A+C\\ A+C & A+C+E \end{array}\right).$$

The DZ twin covariance, for *i* with $T\left(i\right)=T\left(j\left(i\right)\right)=\mathrm{DZ}$, is

(5) $$\mathbb{C}\text{ovDZ}=\mathrm{\mathbb{C}ov}\left(\begin{array}{c} Y_{i}\\ Y_{j\left(i\right)} \end{array}\right)=\left(\begin{array}{cc} A+C+E & A/2+C\\ A/2+C & A+C+E \end{array}\right).$$

For subject pairs involving one or more singletons from different families without twins, (*i*, *j*) with $T\left(i\right)=S$ or $T\left(j\right)=S$, or pairs of unrelated twins, (*i*, *j*) with *j* ≠ *j*(*i*), we have unrelated covariance of

(6) $$\mathbb{C}\text{ovUN}=\mathrm{\mathbb{C}ov}\left(\begin{array}{c} Y_{i}\\ Y_{j} \end{array}\right)=\left(\begin{array}{cc} A+C+E & 0\\ 0 & A+C+E \end{array}\right).$$

To facilitate a general implementation, we re‐write the pair‐wise covariance matrices for MZ twins (4), DZ twins (5) and unrelated subjects (6) as the linear combinations of some known matrices, respectively:

(7) $$\mathbb{C}\text{ovMZ}=\left(\begin{array}{cc} A+C+E & A+C\\ A+C & A+C+E \end{array}\right)=A\left(\begin{array}{cc} 1 & 1\\ 1 & 1 \end{array}\right)+C\left(\begin{array}{cc} 1 & 1\\ 1 & 1 \end{array}\right)+E\left(\begin{array}{cc} 1 & 0\\ 0 & 1 \end{array}\right),$$

(8) $$\mathbb{C}\text{ovDZ}=\left(\begin{array}{cc} A+C+E & A/2+C\\ A/2+C & A+C+E \end{array}\right)=A\left(\begin{array}{cc} 1 & 1/2\\ 1/2 & 1 \end{array}\right)+C\left(\begin{array}{cc} 1 & 1\\ 1 & 1 \end{array}\right)+E\left(\begin{array}{cc} 1 & 0\\ 0 & 1 \end{array}\right),$$

(9) $$\mathbb{C}\text{ovUN}=\left(\begin{array}{cc} A+C+E & 0\\ 0 & A+C+E \end{array}\right)=A\left(\begin{array}{cc} 1 & 0\\ 0 & 1 \end{array}\right)+C\left(\begin{array}{cc} 1 & 0\\ 0 & 1 \end{array}\right)+E\left(\begin{array}{cc} 1 & 0\\ 0 & 1 \end{array}\right),$$

where variance components are extracted as the coefficients. If we denote the vector of variance components *A*, *C*, *E* by ***ρ*** = (*A*, *C*, *E*)^*′*^, a concise notation of the error variance‐covariance matrix **V** is

$$V=\sum_{r=1}^{3}\rho_{r}Q_{r},$$

where **Q**_*r*_ (*r* = 1, 2, 3) is constructed with the use of between‐subject covariances (7), (8), and (9), corresponding to the arrangement of MZ, DZ and singletons in the data vector. The full likelihood and restricted likelihood (ReML) that accounts for nuisance regressors can be found in (Harville, 1977).

### 2.2 Voxel‐wise heritability estimation

For each voxel, we fit the GLM model to the voxel‐wise data from twins and singletons, then estimate the model parameters—variance components, and finally obtain the estimate of heritability. We will first describe our proposed method in detail, and then briefly review other methods/tools that are widely used for heritability estimation.

#### 2.2.1 Linear regression with squared differences

In the 1980s, a simple linear regression method for variance component estimation using squared differences (SqD's) of each subject pair was proposed by Grimes and Harvey (1980). For a sample of *n* subjects, there are (*n* ^2^ − *n*)/2 distinct SqD's. Note that the expectation of a SqD depends on the covariance, that is, $E\left[{\left(A-B\right)}^{2}\right]=V\mathrm{ar}\left(A-B\right)=V\mathrm{ar}\left(A\right)+V\mathrm{ar}\left(B\right)-2C\mathrm{ov}\left(A,B\right)$ for random variables *A* and *B* with $E\left[A\right]=E\left[B\right]$. This allows SqD's to be related to variance parameters in a linear fashion, in particular construction of a linear regression where coefficients correspond to the variance components *A*, *C*, *E* (Grimes & Harvey, 1980; Lindquist, Spicer, Asllani, & Wager, 2012). Grimes and Harvey (1980) used ordinary least squares (OLS), which can produce negative variance component estimates that they simply neglected.

To deal with the non‐negativity problem, Lawson and Hanson (1987) proposed a now ubiquitous non‐negative least squares (NNLS) algorithm. The foundation of this algorithm is the Karush–Kuhn–Tucker (KKT) conditions (Karush, 1939; Kuhn & Tucker, 1951), which were first proposed for more complex nonlinear programming problems. In our case with the linearity assumption, the KKT conditions can be simplified to accelerate the computation. Although other methods had been proposed to solve this non‐negativity problem for large and sparse matrix settings, Luo and Duraiswami (2011) suggested that NNLS still maintained its superiority when small or moderate dense matrices were handled.

While Grimes and Harvey's method specifies a linear regression with the use of (*n* ^2^ − *n*)/2 different observations of SqD's, our modification of this method simplifies the computation such that only (*n*_MZ_ + *n*_DZ_)/2 observations are utilized in computing SqD's. Thus, the integration of the construction of the linear regression model with SqD's and estimating variance‐covariance parameters using NNLS with computational modification yields a novel and fast NNLS regression approach for unknown variance component estimation, entitled “Accelerated Permutation Inference for the ACE model (APACE)”. The method of linear regression model construction with SqD's vary depending on whether subject‐specific covariates are included in the GLM model or not.

##### One sample model

Consider the case when the original GLM (3) is a simple linear regression model with an intercept only:

(10) $$Y=1\beta_{0}+ɛ,$$

where **1** is an all‐ones vector and *β*_0_ denotes the population mean. By the extension of the covariance matrices (4), (5), and (6) and the basic properties of the variance operator, for MZ twin pairs (*i*, *j*(*i*)), $T\left(i\right)=\mathrm{MZ}$, we have

(11) $$E\left[{\left(Y_{i}-Y_{j\left(i\right)}\right)}^{2}\right]=V\mathrm{ar}\left({ɛ}_{i}-{ɛ}_{j\left(i\right)}\right)=2E,$$

for DZ twin pairs (*i*, *j*(*i*)), $T\left(i\right)=\mathrm{DZ}$, we have

(12) $$E\left[{\left(Y_{i}-Y_{j\left(i\right)}\right)}^{2}\right]=V\mathrm{ar}\left({ɛ}_{i}-{ɛ}_{j\left(i\right)}\right)=A+2E,$$

and for unrelated pairs of subjects (*i*, *j*),

(13) $$E\left[{\left(Y_{i}-Y_{j}\right)}^{2}\right]=V\mathrm{ar}\left({ɛ}_{i}-{ɛ}_{j}\right)=2A+2C+2E.$$

The relationships (11), (12), and (13) describe the expected values for all these (*n* ^2^ − *n*)/2 SqD's and specify the mean structure of a linear regression model:

$$E\left[\left(\begin{array}{c} {\left(Y_{1}-Y_{j\left(1\right)}\right)}^{2}\\ \vdots \\ {\left(Y_{n_{\mathrm{MZ}}/2+1}-Y_{j\left(n_{\mathrm{MZ}}/2+1\right)}\right)}^{2}\\ \vdots \\ {\left(Y_{i}-Y_{j}\right)}^{2}\\ \vdots \end{array}\right)\right]=\left(\begin{array}{ccc} 0 & 0 & 2\\ & \vdots & \\ 1 & 0 & 2\\ & \vdots & \\ 2 & 2 & 2\\ & \vdots & \end{array}\right)\left(\begin{array}{c} A\\ C\\ E \end{array}\right),$$

where the first *n*_MZ_/2 rows are the SqD's of MZ twins, the next *n*_DZ_/2 rows are for the DZ twins, and the remaining (*n*^2^ − *n*)/2 − *n*_MZ_/2 − *n*_DZ_/2 rows are for the remaining unrelated subject pairings. We denote this as

(14) $$E\left[D\right]=Z\rho ,$$

where, **D** is an (*n* ^2^ − *n*)/2 × 1 SqD vector of observations, **Z** is an (*n* ^2^ − *n*)/2 × 3 design matrix, and *ρ* is the unknown variance parameter vector.

##### Multiple linear regression

Now suppose that the GLM (3) is a multiple regression model containing an regression intercept and multiple covariates, expressed as

(15) $$Y=1\beta_{0}+X_{1}\beta_{1}+\ldots +X_{p-1}\beta_{p-1}+ɛ,$$

where the *n*‐vectors **X**_1_, … , **X**_*p* − 1_ are regressors, each associated with one of the *p* − 1 covariates, and *β*_1_, …, *β*_*p* − 1_ are the corresponding regression coefficients. The parameters *β* = (*β*_0_, …, *β*_*p* − 1_)^*′*^ are not of interest and we treat them as nuisance parameters in variance component analysis. If we estimate *β* using OLS with an expression of ${\hat{\beta }}_{\mathrm{OLS}}={\left(X\prime X\right)}^{-1}X^{\prime }Y$, where **X** = (**1**, **X**_1_,  … , **X**_*p* − 1_) is the complete design matrix, the resulting OLS residuals are

(16) $$e=Y-X{\hat{\beta }}_{\mathrm{OLS}}=\left(I-X{\left(X\prime X\right)}^{-1}X^{\prime }\right)Y.$$

Denote the residual projection matrix by **R** = **I** − **X**(**X** ′ **X**)^−1^**X**^′^, and the OLS residual vector **e** = **RY** follows a normal distribution with mean $E\left[e\right]=0$ and variance ℂov(**e**) = **RVR**, that is, **e**~ℕ(**0**, **RVR**), where the projection matrix **R** projects the unobservable error vector *ɛ* to its estimate **e** that is orthogonal to the space spanned by the columns of the design matrix **X**. When the sample size *n* is large enough, the error vector *ɛ* can be well approximated by the residual vector **e**. We assume that the correlation induced by removing covariates and mean centering is negligible when compared with variance components, that is, **RVR** ≈ **V**, which is a reasonable assumption for sufficient *n*.

Here the variance components are derived in terms of nuisance‐free errors:

$$E\left[{\left(e_{i}-e_{j\left(i\right)}\right)}^{2}\right]=V\mathrm{ar}\left(e_{i}-e_{j\left(i\right)}\right)\approx 2E$$

for MZ twin pairs,

$$E\left[{\left(e_{i}-e_{j\left(i\right)}\right)}^{2}\right]=V\mathrm{ar}\left(e_{i}-e_{j\left(i\right)}\right)\approx A+2E$$

for DZ twin pairs, and

$$E\left[{\left(e_{i}-e_{j}\right)}^{2}\right]=V\mathrm{ar}\left(e_{i}-e_{j}\right)\approx 2A+2C+2E.$$

for the remaining unrelated subject pairs. Therefore, the derived linear regression model with SqD's can be analogously denoted as

(17) $$E\left[D\right]\approx Z\rho .$$

##### Computational simplification

For large *n*, the (*n* ^2^ − *n*)/2 rows of the SqD data and design matrix is unwieldy. Hence we modify how we compute estimates $\hat{\rho }={\left(Z^{\prime }Z\right)}^{-1}Z^{\prime }D$. First, **Z**^′^**Z** is directly found as

$$Z^{\prime }Z=\left(\begin{array}{ccc} n_{\mathrm{DZ}}/2+4n_{\mathrm{otw}} & 4n_{\mathrm{otw}} & n_{\mathrm{DZ}}+4n_{\mathrm{otw}}\\ 4n_{\mathrm{otw}} & 4n_{\mathrm{otw}} & 4n_{\mathrm{otw}}\\ n_{\mathrm{DZ}}+4n_{\mathrm{otw}} & 4n_{\mathrm{otw}} & 2\left(n^{2}-n\right) \end{array}\right),$$

where *n*_otw_ = (*n*^2^ − *n*)/2 − *n*_MZ_/2 − *n*_DZ_/2. Next, observe that
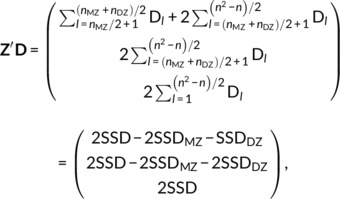
where, D_*l*_ is the *l*^th^ element of **D**, $\mathrm{SSD}=\sum_{l=1}^{\left(n^{2}-n\right)/2}D_{l}$ is the sum of all squared differences, SSD_MZ_ is the sum of *n*_MZ_/2 squared differences for MZ, and SSD_DZ_ is the sum of *n*_DZ_/2 squared differences for DZ. A fundamental result (see Appendix A) shows that SSD is just a multiple of the sample variance:

$$\mathrm{SSD}=\left(n^{2}-n\right)s^{2}\left(Y\right),$$

where $s^{2}\left(Y\right)=\sum_{i}^{n}{\left(Y_{i}-\bar{Y}\right)}^{2}/\left(n-1\right)$. With the nuisance variables, we approximate this sum with the residual variance from the GLM (3), that is,

$$\mathrm{SSD}\approx \left(n^{2}-n\right){\hat{\sigma }}^{2},$$

where ${\hat{\sigma }}^{2}=e^{\prime }e/\left(n-p\right)$ is the unbiased estimator for the phenotypic variance *σ* ^2^. With simulations, we verified that estimating SSD with this residual variance had a negligible impact on parameter estimates.

##### Non‐negative least squares

Our APACE method proceeds by applying the NNLS algorithm to the linear regression model with SqD's (10) or (15) for unknown variance component estimation; precisely, we seek

$$\min_{\rho }f\left(\rho \right)\;s.t.\rho \geq 0,$$

where *f*(***ρ***) = ‖**Z*ρ*** − **D**‖^2^/2 is the objective function to be minimized. KKT conditions provide the necessary conditions for this optimization problem: If *ρ* ^*^ is the local minimizer of *f*(*ρ*) satisfying the inequality constraint *ρ* ≥ 0, then the following conditions hold:

$$\nabla f{\left(\rho^{*}\right)}^{\prime }\rho^{*}=0,\;\nabla f\left(\rho^{*}\right)\geq 0,\;\rho^{*}\geq 0,$$

where, the gradient vector is ∇*f*(***ρ***) = **Z**^′^(**Z*ρ*** − **D**) (Karush, 1939; Kuhn & Tucker, 1951). As **Z**^′^(**Z*ρ*** − **D**) = **0** corresponds to the least squares normal equations, for any **Z**, **D**, and *ρ* found by OLS, the first two conditions are trivially satisfied, and hence, only the third condition needs to be checked. The NNLS algorithm proceeds by using OLS to estimate *ρ* and checking for negative elements. If a negative element is found, it is set to zero, effectively dropping that column from the **Z**, and OLS is re‐fit.

##### Algorithm simplification

The NNLS algorithm can be further modified and simplified for the computation in ACE modeling since there are only three parameters *A*, *C*, *E* in total. There are only a total of 2^3^ − 1 = 7 possible models that can arise under NNLS, but we require the unique environmental factor *E* to always be present due to the unavoidable measurement error or noise. Thus we only need to consider four possible models E, AE, CE, and ACE with ***ρ***_E_ = (0, 0, *E*)^′^,  ***ρ***_AE_ = (*A*, 0, *E*)^′^,  ***ρ***_CE_ = (0, *C*, *E*)^′^ and ***ρ***_ACE_ = (*A*, *C*, *E*)^′^ representing the vectors of unknown parameters, respectively.

Since the space of possible models is so small, we can enumerate and evaluate these four models. We first fit the full ACE model, and if all the *A*, *C*, *E* parameters are non‐negative, this estimate ${\hat{\rho }}_{\mathrm{ACE}}$ is used; otherwise, the remaining estimates ${\hat{\rho }}_{E},\;{\hat{\rho }}_{\mathrm{AE}}$, and ${\hat{\rho }}_{\mathrm{CE}}$ corresponding to the three restricted models are computed. Among the optional models with valid estimates (i.e., all non‐negative), the best fitting model is selected; as valid AE and CE models will always explain more variability than an E model, these are next selected when available. If both AE and CE models have valid estimates, we have two methods to assess the model fit. The model with the smallest residual sum of squares, that is, ${\left(D-Z\hat{\rho }\right)}^{\prime }\left(D-Z\hat{\rho }\right)$, can be selected, which is our default APACE setting. Alternatively, we can return to the original GLM model (3) and select the model with the higher ReML log‐likelihood for APACE‐computed ${\hat{\rho }}_{\mathrm{AE}}$ and ${\hat{\rho }}_{\mathrm{CE}}$. This process to choose between the two models is equivalent to using a model selection method based on Akaike's information criterion or Bayesian information criterion.

##### Likelihood ratio test

Tests on parameter estimates are performed as usual, with a likelihood ratio test (LRT) comparing the fitted model (alternative model) H_1_: *A* > 0 to the null model H_0_: *A* = 0, that is, the full ACE model is compared to the nested CE model, or AE to E. The LRT statistic is defined as

$$T=-2\times \left[\ell \left({\hat{\rho }}_{0}|Y\right)-\ell \left({\hat{\rho }}_{1}|Y\right)\right],$$

where, ${\hat{\rho }}_{0}$ and ${\hat{\rho }}_{1}$ are parameter estimates derived from the null and alternative models, respectively, and $\ell \left(\hat{\rho }|Y\right)$ is the ReML log‐likelihood given observations **Y**. Under the assumption of normality, the likelihood function can be analytically computed (see for example, Harville, 1977). Wilks' theorem states that under certain regularity conditions, the null distribution of the LRT statistic for comparing nested models (e.g., CE vs. ACE) converges to a chi‐squared distribution as *n* → *∞* (Wilks, 1938). In particular, *T* is usually regarded to asymptotically follow a chi‐squared distribution with 1 degree of freedom, that is, $\chi_{1}^{2}$, based on Wilks' theorem. However, the null value of the variance parameter *A* lies on the boundary of the parameter space and thus the asymptotic sampling distribution of this LRT statistic, under H_0_, is a half‐half mixture of chi‐squared distributions, that is, $\chi_{0}^{2}/2+\chi_{1}^{2}/2$, instead of a commonly used standard chi‐square distribution, that is, $\chi_{1}^{2}$ (Dominicus, Skrondal, Gjessing, Pedersen, & Palmgren, 2006; Self & Liang, 1987; Zhang & Lin, 2008).

Given the asymptotic null distribution of the LRT statistic, the theoretical *p*‐value can be easily calculated. Obtaining a *p*‐value less than a given significance level *α*, which is typically a small number (e.g., *α* = 0.05), suggests that there is significant evidence against the null hypothesis and the null hypothesis should be rejected at level *α*. We note that when data is non‐Gaussian, the LRT computed under the normality assumption can be inaccurate and the use of Wilk's theorem in approximating its null distribution might lead to invalid results, which will be investigated in our simulation studies. We therefore compute both the asymptotic theoretical *p*‐value and the permutation‐based *p*‐value based on the empirical distribution of the LRT statistic (see below).

#### 2.2.2 Existing methods

Several approaches have been proposed for the analysis of heritability, and we briefly introduce these existing approaches in this section.

##### Falconer's method

The heritability method due to Falconer and Mackay (1996) is based on moment matching of intraclass correlation coefficients between MZ twins (*r*_MZ_) and DZ twins (*r*_DZ_):

$$\left\{\begin{array}{l} E\left[r_{\mathrm{MZ}}\right]=\frac{A+C}{A+C+E},\\ E\left[r_{\mathrm{DZ}}\right]=\frac{A/2+C}{A+C+E}. \end{array}\right.$$

Solving for narrow‐sense heritability (2), these equations give Falconer's heritability estimator:

$${\hat{h}}_{F}^{2}=\max\left(0,2\left(r_{\mathrm{MZ}}-r_{\mathrm{DZ}}\right)\right).$$

This method has been widely used and is the simplest way to estimate heritability (Falconer & Mackay, 1996). However, methods of moments estimators can perform poorly relative to optimal maximum likelihood estimators (Nichols, Friston, Roiser, & Viding, 2009). We consider this in a small set of simulations, described below.

The null hypothesis of zero heritability can be tested by comparing the MZ and DZ correlation coefficients after Fisher's variance‐stabilising transformation. For MZ, Fisher's transformation is

$$z_{\mathrm{MZ}}=\frac{1}{2}\log\left(\frac{1+r_{\mathrm{MZ}}}{1-r_{\mathrm{MZ}}}\right),$$

which is approximately normally distributed with mean

$$E\left[z_{\mathrm{MZ}}\right]=\frac{1}{2}\log\left(\frac{1+\rho_{\mathrm{MZ}}}{1-\rho_{\mathrm{MZ}}}\right)$$

and variance

$$V\mathrm{ar}\left(z_{\mathrm{MZ}}\right)=\frac{1}{n_{\mathrm{MZ}}/2-3},$$

where *ρ*_MZ_ is the true population correlation coefficient; likewise for *z*_DZ_. To test for the equality of *r*_MZ_ and *r*_DZ_, that is, zero heritability, we can use the test statistic

$$T_{F}=\frac{z_{\mathrm{MZ}}-z_{\mathrm{DZ}}}{\sqrt{\frac{1}{n_{\mathrm{MZ}}/2-3}+\frac{1}{n_{\mathrm{DZ}}/2-3}}}1_{\left\{h_{F}^{2}>0\right\}},$$

where **1**_{⋅}_ is the indicator function. Without the positivity constrain, this test statistic would be asymptotically distributed as a standard normal distribution under H_0_ (Sakaori, 2002). Considering the positivity constraint, we consider the null distribution to be a half‐half mixture of a point mass at zero (a.k.a. $\chi_{0}^{2}$) and a half normal distribution.

##### Bayesian ReML

Statistical parametric mapping (SPM) software (http://www.fil.ion.ucl.ac.uk/spm) provides a general framework for variance component model estimation in a Bayesian setting, and, as a special case, can implement the ACE model. Based on preliminary studies (Nichols et al., 2009), we show that this Bayesian approach produces heritability estimates with lower bias and variance than Falconer's method. SPM's Bayesian ReML uses a log Gaussian prior on variance parameters, ensuring non‐negative variance parameter estimates. We set the prior mean and variance of the log variance parameters to be $hE=\log\left(V\mathrm{ar}\left(Y\right)\right)-1$ and *h*C = exp(8) to produce uninformative priors. We considered perturbations of these settings but simulations found that these priors were best in terms of estimation accuracy and power (not shown). SPM uses the expectation‐maximization (EM) algorithm to iteratively search for the maximum a posteriori estimates of the parameters in the log space (Friston et al., 2002 b).

##### Structural equation modeling

**Figure 1:**
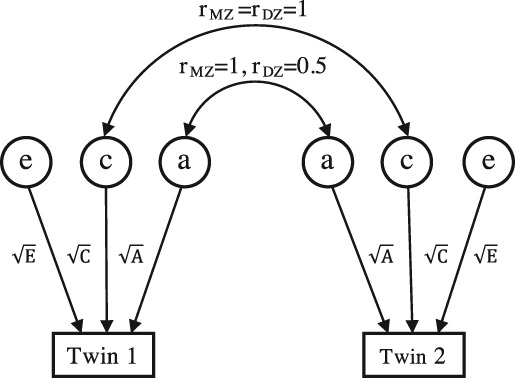
Path diagram for the univariate ACE twin model

The freely available R package “OpenMx” (http://openmx.psyc.virginia.edu) offers a structural equation SEM framework to allow flexible model definition and parameter estimation for variance components, both of which are commonly used in analysing genetic data for heritability inference. The SEM ACE model for univariate twin data can be displayed as a path diagram, shown in Figure 1, where the influence caused by the latent variables *a*, *c*, and *e* can be described by the path coefficients $\sqrt{A},\sqrt{C}$ and $\sqrt{E}$, respectively (Rijsdijk & Sham, 2002). According to path tracing rules, the covariance matrices for MZ and DZ twin pairs are

$$\begin{array}{l} \mathbb{C}\text{ovMZ}=\left(\begin{array}{cc} A+C+E & A+C\\ A+C & A+C+E \end{array}\right),\\ \mathbb{C}\text{ovDZ}=\left(\begin{array}{cc} A+C+E & A/2+C\\ A/2+C & A+C+E \end{array}\right), \end{array}$$

which have the same structure as matrices (4) and (5). The goodness of fit of this model is also measured using the maximum likelihood criterion (Rijsdijk & Sham, 2002). However, there exist some drawbacks of this SEM approach employed in OpenMx for imaging data analysis. The goodness‐of‐fit LRT statistic asymptotically follows a mixture of chi‐square distributions (Dominicus et al., 2006; Self & Liang, 1987; Zhang & Lin, 2008), but we can observe that OpenMx incorrectly uses a single standard chi‐square distribution with 1 degree of freedom (Rijsdijk & Sham, 2002). For neuroimaging, a relative weakness of OpenMx is lack of direct tools for operating with neuroimaging data.

##### Solar

The sequential oligogenic linkage analysis routines (SOLAR) software is designed for the investigation of genetic effects in imaging genetics studies (Almasy & Blangero, 1998; Koran et al., 2014). In addition, the SOLAR package is capable of estimating heritability with the data from diverse family structures. SOLAR uses maximum likelihood to estimate the variance parameters, *A*, *C*, *E*. To test the null hypothesis of zero heritability, the LRT test statistic, which is asymptotically distributed as a mixture of chi‐square distributions, can be calculated (Almasy & Blangero, 1998). While SOLAR itself cannot read brain imaging data, the related software SOLAReclipse (https://www.nitrc.org/projects/se_linux) can read and write neuroimaging data.

### 2.3 Permutation inference

Permutation testing is a nonparametric technique that makes minimal assumptions about the data. With a few simple assumptions like exchangeability of the observed data under the null hypothesis, the nonparametric permutation test is conceptually simple and theoretically intuitive (Nichols & Hayasaka, 2003; Nichols & Holmes, 2001). When the null hypothesis is true, the data will exhibit the feature of exchangeability, allowing permutation, re‐fitting the model and computation of the test statistic. With multiple permutations, an empirical null distribution can be constructed and critical thresholds and *p*‐values computed. This approach has become feasible owing to the widespread availability of powerful computers.

Applying variance component inference approach voxel‐by‐voxel yields a test statistic image. For each voxel, if the null hypothesis of no heritability, that is, H_0_: *h* ^2^ = 0, is assumed to be true, MZ and DZ twin pairs become exchangeable, allowing $N_{p}=\left(\begin{array}{c} \left(n_{\mathrm{MZ}}+n_{\mathrm{DZ}}\right)/2\\ n_{\mathrm{MZ}}/2 \end{array}\right)$ possible permutations of MZ and DZ labels on twin pairs. In the presence of nuisance covariates **X**, residuals are permuted (here, as stated above, we assume that the residuals **e** approximate the true errors *ɛ*). Crucially, twin pairs stay linked, preserving any common environment effects. As even moderate sample sizes can yield *N*_*p*_ too large to allow computing all permutations, in this case, an approximate or Monte Carlo permutation test is conducted for a smaller number of permutations, say *N*_*p*_ = 1,000, based on a random subsample of all permutations (Nichols & Holmes, 2001).

In order to resolve the multiple comparisons problem and strictly control false positives over the whole volume of regions of interest (ROI's) or voxels simultaneously, a permutation test can also be used to compute inferences corrected for the family‐wise error (FWE). FWE‐corrected *p*‐values are found by considering the null distribution of maximum test statistics (Nichols & Holmes, 2001). With a permutation test, we obtain FWE‐corrected *p*‐values on peak height (voxel‐wise test statistic value) for voxel‐wise inference, and cluster size (number of voxels involved in a cluster after thresholding) and cluster mass (sum of voxel‐wise test statistic values of all voxels within a cluster after thresholding) for cluster‐level inference. Hence, this permutation approach can be further partitioned into two parts: voxel‐wise single threshold test and cluster‐wise supra‐threshold tests.

#### 2.3.1 Voxel‐wise single threshold test

Let *π* = 1, …, *N*_*p*_ be the permutations considered including correctly labeled data. At a single ROI/voxel, the permutation test produces a level *α* threshold by finding the ⌊*αN*_*p*_⌋ + 1 largest value of the null distribution; *p*‐values are computed as the proportion of null distribution values as large as or larger than the observed test statistic.

To control the FWE, the relevant null distribution is that of the maximum test statistic. That is, the level‐*α* FWE threshold $T_{\alpha }^{\mathrm{FWE}}$ is the ⌊*αN*_*p*_⌋ + 1 largest value of the maximum distribution, and FWE‐corrected *p*‐value, denoted by $p_{T}^{\mathrm{FWE}}$, for any given test statistic *T*_0_ is likewise the proportion of permutation maximum distribution values equaling or exceeding that value (Nichols & Holmes, 2001):

$$p_{T}^{\mathrm{FWE}}=\frac{\#\left\{T_{\pi }^{\max}\geq T_{0}\right\}}{N_{p}},$$

where $T_{\pi }^{\max}$ is element *π* of the maximum distribution.

#### 2.3.2 Cluster‐wise supra‐threshold tests

The significance of supra‐threshold cluster tests can be assessed by the spatially informed cluster statistics, such as cluster size and cluster mass. A preselected cluster‐forming threshold *u*, which can be expressed as a *p*‐value using the sampling distribution of the test statistic, is applied to the derived test statistic image to threshold test statistic values and form supra‐threshold clusters. A cluster size *K*, the count of voxels in a cluster, or a cluster mass *M*, the sum of test statistic values exceeding *u*, can be computed. As clusters are random in number and location, uncorrected *p*‐values can be found but reflect an assumption of homogeneity over space and typically are not computed.

Computation of FWE‐corrected inferences proceeds as with the single‐threshold test. The ⌊*αN*_*p*_⌋ + 1 largest element of the null distribution of maximum size (or mass) defines a critical level‐*α* FWE‐corrected threshold $K_{\alpha }^{\mathrm{FWE}}$ (or $M_{\alpha }^{\mathrm{FWE}}$). The associated FWE‐corrected *p*‐values are

$$p_{K}^{\mathrm{FWE}}=\frac{\#\left\{K_{\pi }^{\max}\geq K_{0}\right\}}{N_{p}},$$

$$p_{M}^{\mathrm{FWE}}=\frac{\#\left\{M_{\pi }^{\max}\geq M_{0}\right\}}{N_{p}},$$

for cluster statistics of size and mass, respectively, where $K_{\pi }^{\max}$ (or $M_{\pi }^{\max}$) is element *π* of the maximum cluster size (or mass) distribution.

## 3 SIMULATION STUDIES

In this section, univariate simulation analysis is conducted to compare our newly proposed voxel‐wise heritability estimation methods with existing methods in terms of prediction accuracy, validity, sensitivity, and the overall computation time for different variance component settings. The ROC‐based simulation studies generate 2D image data for power evaluation and comparison between the voxel‐ and cluster‐wise heritability inference approaches for various settings.

### 3.1 Univariate simulation evaluations

We use Monte Carlo simulations to evaluate our proposed linear regression methods, LR‐SqD Perm (LR‐SqD using empirical *p*‐value based on 1,000 permutations), LR‐SqD (LR‐SqD using asymptotic *p*‐value) and LR‐SqD ReML, and existing methods including Falconer's method, Bayesian ReML in SPM, SEM in OpenMx, and SOLAR.

#### 3.1.1 Simulation setting

**Figure 2:**
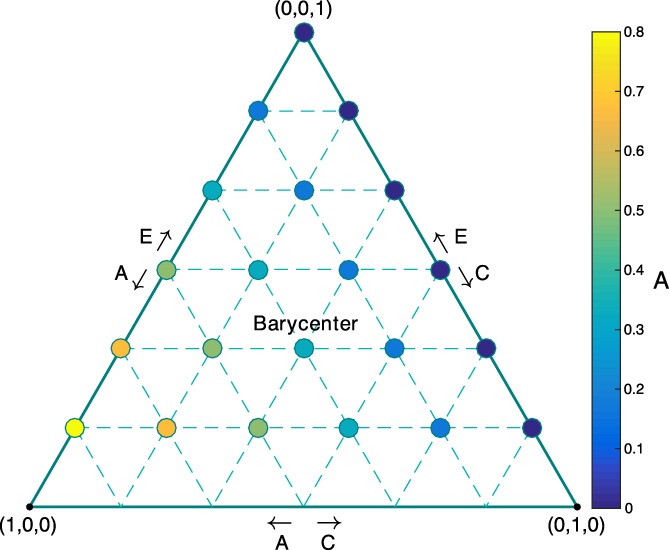
Parameter space with various (*A*, *C, E*) parameter settings using Barycentric coordinates with barycenter (*A*, *C*, *E*) = (1/3, 1/3, 1/3). The large equilateral triangle (solid line) shows the ACE parameter space with the constraints *A* + *C* + *E* = 1, *A, C*, *E* ≥ 0, where the vertices (filled circles) represents the selected *A*, *C*, *E* parameter settings shown in Table 1. The color of each vertex indicates the value of *A* [Color figure can be viewed at http://wileyonlinelibrary.com]

The parameter settings shown in Table 1 are motivated as follows. If we create a 3D Cartesian coordinate system with x, y and z axes representing the possible values for *A*, *C*, *E*, then the 3D parameter space can be visualized as an equilateral triangle in 2D space utilizing a Barycentric coordinate system, as shown in Figure 2. Since *E* ≫ *max* (*A*, *C*) usually holds in practice, we choose 15 possible sets of *A*, *C*, *E* from the upper part of this parameter space satisfying *E* ≥ 1/3. Note that we assign values of *A*, *C*, *E* such that *A* + *C* + *E* = 1, meaning *A* is directly interpretable as *h* ^2^. That is, the value of *A* is exactly *h* ^2^. However, we still use

$${\hat{h}}^{2}=\frac{\hat{A}}{\hat{A}+\hat{C}+\hat{E}}$$

during computation to account for the genetic random variation in total variance.

**Table 1:** Fifteen *A*, *C*, *E* parameter settings

	*A*	*C*	*E*
Complete null	0	0	1
Only Env.	0	1/6	5/6
	0	1/3	2/3
	0	1/2	1/2
	0	2/3	1/3
Only Gen.	1/6	0	5/6
	1/3	0	2/3
	1/2	0	1/2
	2/3	0	1/3
Gen. and Env.	1/6	1/6	2/3
	1/3	1/6	1/2
	1/6	1/3	1/2
	1/2	1/6	1/3
	1/3	1/3	1/3
	1/6	1/2	1/3

For balanced design with an equal number of subjects for each group (MZ and DZ twins), three samples are considered with the size of *n* = 100, 300, 1,000. For instance, the sample of 100 subjects is comprised of 25 MZ twin pairs (50 subjects) and 25 DZ twin pairs (50 subjects). For the unbalanced design, the sample size is fixed as *n* = 300, and we consider the MZ:DZ ratios being 1:4 and 4:1, that is, *n* = 60 + 240 and *n* = 240 + 60.

Apart from the Gaussian random error, we also take into account the case where the error term is not normally distributed. Here we consider a log‐normally distributed unique environmental random noise. The sample considered is balanced with the size of *n* = 300, that is, the number of MZ and DZ twin pairs is identical.

In total, 5 samples with balanced/unbalanced design and Gaussian/non‐Gaussian random error, along with 15 (*A*, *C*, *E*) parameter settings, lead to totally 90 simulation settings. For each setting, we consider both the one sample model (10) and multiple linear regression (15) to fit SqD's. For the one sample model, no covariates are included in the model (3) and the design matrix is an all‐ones vector. For multiple linear regression, age, sex, the interaction between age and sex and a standard normally distributed continuous variable are included as covariates. The regressors are simulated using Matlab. **X** thus has five columns, in which the first column is an all‐ones vector for the intercept and the remaining are randomly generated vectors approximating the four covariates. Results are based on 1,000 realisations.

The mean squared error (MSE) is calculated to compare 6 estimation methods including LR‐SqD, LR‐SqD ReML, Falconer's method, Bayesian ReML in SPM, SEM in OpenMx, and SOLAR. For each of all seven testing (inference) methods considered (LR‐SqD Perm, LR‐SqD, LR‐SqD ReML, Falconer's method, Bayesian ReML in SPM, SEM in OpenMx and SOLAR), we compute false positive rate (FPR), statistical power, and overall running time. As the one sample model and multiple linear regression model have qualitatively similar results, we will only report the results obtained from multiple linear regression.

#### 3.1.2 Comparison results

##### Accuracy and precision

**Figure 3:**
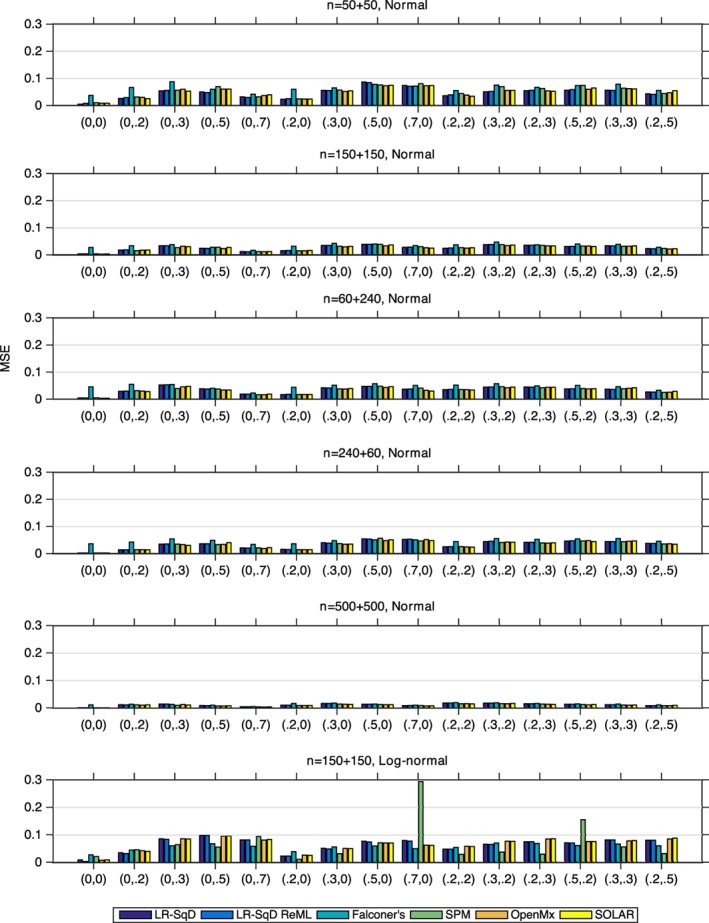
The MSE comparison of LR‐SqD, LR‐SqD ReML, Falconer's method, Bayesian ReML in SPM, SEM in OpenMx, and SOLAR, based on 1,000 realizations (Rows 1–5 for Gaussian error and Row 6 for non‐Gaussian error). Comma ordered pairs on *x*‐axis correspond to the rounded parameter values of *A* and *C*, that is, (*A*, *C*); see Table 1 and Figure 2 for exact parameter settings used [Color figure can be viewed at http://wileyonlinelibrary.com]

The MSE comparison of six methods is shown in Figure 3, which shows that the two linear regression methods, LR‐SqD and LR‐SqD ReML, have MSE virtually identical to each other. For the first five rows with Gaussian noise, with the exception of Falconer's method, which generally has markedly worse MSE, all of the methods exhibit roughly comparable MSE performance. For the sixth row with non‐Gaussian noise, the MSE of all methods is larger than that for Row 2 for nearly all parameter settings, and Falconer's method works sometimes better and sometimes worse than the other methods.

##### Specificity and statistical sensitivity

**Figure 4:**
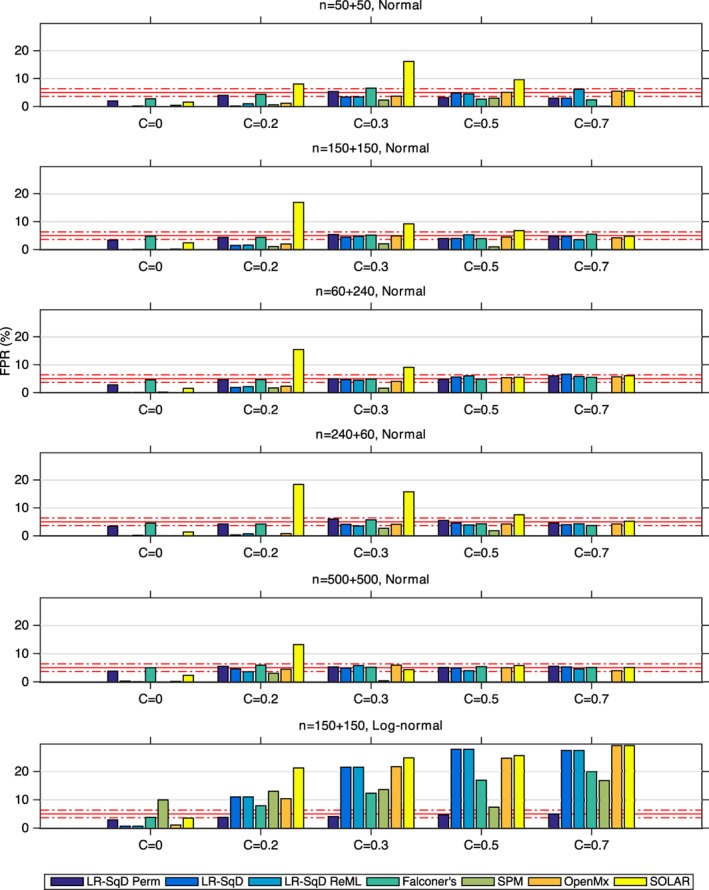
The comparison of the estimated false positive rate (FPR) with a nominal level *α* = 0.05 for true null hypothesis (H_0_: *h* ^2^ = 0, that is, *A* = 0), among LR‐SqD perm using 1,000 permutations, LR‐SqD, LR‐SqD ReML, Falconer's method, Bayesian ReML in SPM, SEM in OpenMx, and SOLAR, based on 1,000 realisations (Rows 1–5 for Gaussian error and Row 6 for non‐Gaussian error). The *x*‐axis represents the rounded values of *C*. The two red dash‐dotted lines show the lower and upper bounds of the 95% binomial proportion confidence interval. The FPR should be 0.05, but its estimates can vary within the 95% binomial proportion confidence interval [0.0365, 0.0635] for 1,000 simulations [Color figure can be viewed at http://wileyonlinelibrary.com]

Figure 4 shows the specificity comparison of the seven testing methods at a nominal significance level *α* = 0.05 (under the null hypothesis of no heritability). With Gaussian noise (Rows 1–5), when the common environment effect *C* is also zero, all methods are highly conservative except LR‐SqD Perm and Falconer's, both of which are close to exact for sufficient sample sizes. In the presence of *C* > 0, the methods are generally valid but SOLAR struggles, having inflated FPR. We believe this is due to convergence problems for small samples. For the non‐Gaussian case (Row 6), we note that all methods are invalid with inflated FPR except LR‐SqD Perm, which does not rely on the assumption of normality.

**Figure 5:**
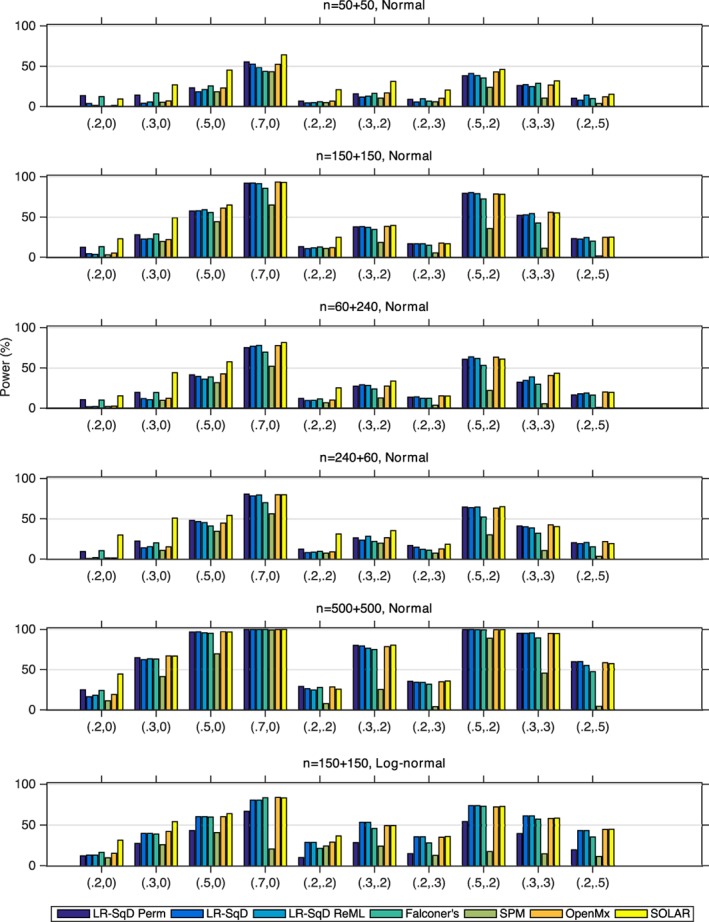
The statistical power comparison with a nominal level *α* = 0.05 for false null hypothesis (H_0_: *h* ^2^ = 0) of LR‐SqD Perm using 1,000 permutations, LR‐SqD, LR‐SqD ReML, Falconer's method, Bayesian ReML in SPM, SEM in OpenMx, and SOLAR, based on 1,000 realizations (Rows 1–5 for Gaussian error and Row 6 for non‐Gaussian error). Comma ordered pairs on *x*‐axis correspond to the rounded parameter values of (*A*, *C*) with *A* > 0; see Table 1 and Figure 2 for exact parameter settings used [Color figure can be viewed at http://wileyonlinelibrary.com]

Figure 5 plots the power results. In the case of Gaussian noise, if we set aside SOLAR's results that must be interpreted in light of its inflated FPR, we find our linear regression methods using asymptotic theoretical *p*‐value and OpenMx have comparable power. LR‐SqD Perm using permutation‐based empirical *p*‐values has the largest power in almost all settings, particularly for small values of heritability and zero *C* effect. SPM's ReML method is generally less powerful. Falconer's method is around the linear regression methods using asymptotic *p*‐value and OpenMx, sometimes more, sometimes less powerful. In the case of log‐normally distributed random error (Row 6), we only consider LR‐SqD Perm with controlled FPR. As expected, the power is generally lower for all settings when compared with the Gaussian case with *n* = 150 + 150 (Row 2).

##### Running time comparison

**Figure 6:**
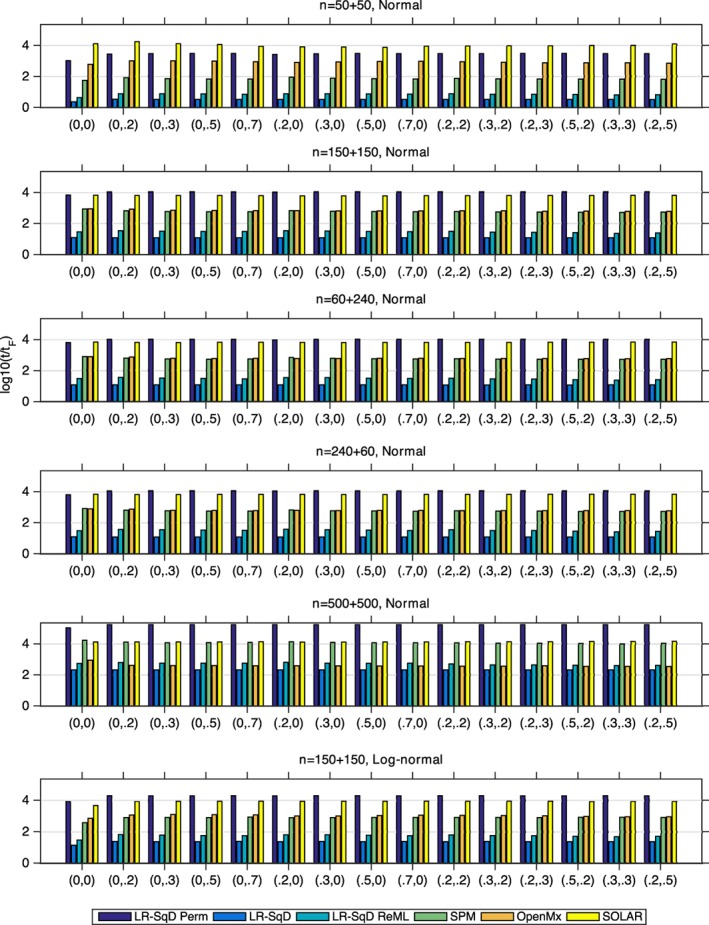
The relative running time comparison after base‐10 log transformation, denoted by log_10_(*t*/*t*_F_) for LR‐SqD Perm using 1,000 permutations, LR‐SqD, LR‐SqD ReML, Bayesian ReML in SPM, SEM in OpenMx, and SOLAR, based on 1,000 realizations, where *t*_F_ denotes the running time for Falconer's method, that is, log_10_(*t*/*t*_F_) = 0 for Falconer's method (Rows 1–5 for Gaussian error and Row 6 for non‐Gaussian error). Comma ordered pairs on *x*‐axis correspond to the rounded parameter values of (*A*, *C*); see Table 1 and Figure 2 for exact parameter settings used [Color figure can be viewed at http://wileyonlinelibrary.com]

We evaluated the relative running time (relative to the running time of Falconer's method) for completion of 1,000 simulated datasets (see Figure 6). The computational performance comparing six methods reveals that Falconer's method and the linear regression methods with SqD's using asymptotic theoretical *p*‐values (i.e., LR‐SqD and LR‐SqD ReML) always outperform other iterative methods including Bayesian ReML in SPM, SEM in OpenMx, and SOLAR. For each simulation setting, the overall computation time of all simulations for those noniterative methods is far smaller than the other iterative methods. The LR‐SqD Perm, based on 1,000 permutations, has running time comparable to, or even longer than, those iterative methods. Nonetheless, applying parallelization by running multiple jobs in parallel can help reduce the overall computation time.

### 3.2 ROC‐based power evaluation

To evaluate the sensitivity of the voxel‐ and cluster‐wise heritability inference approaches described in Section 2.3, we conduct a receiver operating characteristic (ROC) analysis.

#### 3.2.1 Simulation setting

In this set of simulations, we use *n* = 20, 60, 100, using only twins and equal number of MZ and DZ pairs. The signal is generated with four parameter settings shown in Table 2 consisting of different values of heritability and shared environmental variance.

**Table 2:** Four parameter settings of heritability *h* ^2^ and parameters *A*, *C*, *E*

	*h* ^2^	*A*	*C*	*E*
No heritability	0	0	0	1
(*h* ^2^ = 0)				
Positive heritability	1/3	1/3	0	2/3
(*h* ^2^ > 0)	1/2	1/2	1/6	1/3
	2/3	2/3	0	1/3

**Figure 7:**
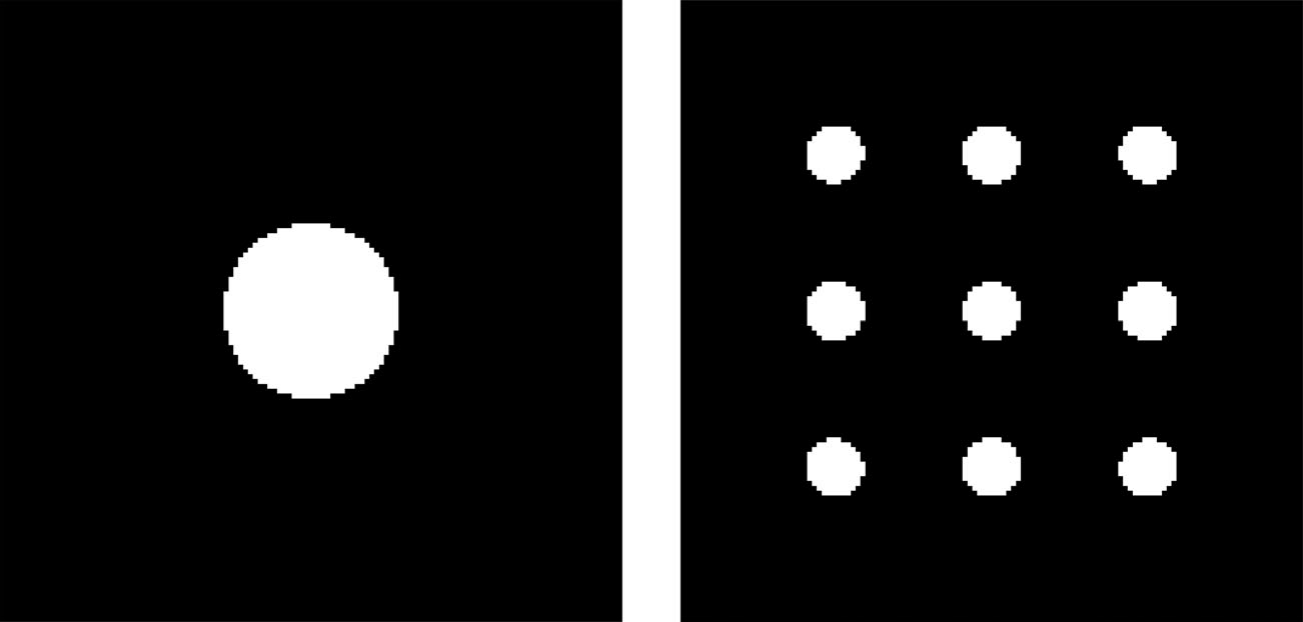
Illustration of the 2D simulated signal shapes. Focal signal (left) with one large circle in the middle; distributed signal (right) with nine identical circular regions

The simulated images are 2D, with 128 × 128 pixels. Two spatial configurations of signal were considered, a single large region or nine separate regions, having similar number of signal pixels (1,020 vs. 1,024); see Figure 7. The set of *N*_I_ images were first created by filling each pixel with i.i.d. standard Gaussian noise, and then inducing the signal with the Cholesky decomposition of the desired variance/covariance structure. Spatial Gaussian smoothing kernels with full width at half maximum (FWHM) of 0, 1.5, 3, or 6 pixels were applied to blur these images in order to accommodate spatial dependence across neighbouring voxels. A total of *N*_I_ = 1000 images were generated for each simulation setting.

#### 3.2.2 ROC analysis

The ROC curves plot true positive rate (TPR; *y*‐axis) against FPR with varying threshold levels. A standard ROC analysis is suitable for only a single outcome, while we have 128^2^ outcomes. Hence we use an alternative, free‐response ROC approach (Chakraborty & Winter, 1990). As described in (Smith & Nichols, 2009), a free‐response ROC, consisting of a *y*‐axis representing the probability of true detection, averaged over pixels with *A* > 0, and an *x*‐axis representing the probability of any false detections, is deployed.

We summarize the ROC curve by a normalized area under the curve (AUC), with a larger value for better performance. Since we are mostly concerned about FPR values between 0 and 0.05, corresponding to a family‐wise error rate of 5%, the normalised AUC is 20 × AUC for FPR < 0.05, maintaining a“perfect” AUC of 1. We calculate this free‐response ROC curve for both voxel‐ and cluster‐wise inferences. For cluster‐wise inference, we set the voxel‐level cluster‐forming threshold to *α* = 0.05 or LRT statistic value *u*_*α*_ = 2.71.

For clarity, the exact steps in this ROC calculation are as follows.

1. Generate *N*_I_ = 1000 i.i.d. 2D smoothed null images with standard Gaussian random noise, where (*A*, *C*, *E*) = (0, 0, 1), and the corresponding smoothed heritability signal images, where the signal were generated with (*A*, *C*, *E*) as per one configuration in Table 2, and one of two spatial configurations in Figure 7.
2. For each image, estimate heritability pixel‐by‐pixel and create the LRT test statistic image.
3. *Voxel‐wise inference*. Apply a large number of predefined grids of thresholds to the LRT test statistic image, obtain the supra‐threshold pixels, and then calculate family‐wise FPR and TPR for each of these threshold levels, obtaining FPR from noise‐only image and TPR from the *A* > 0 pixels in signal images.
*Cluster‐wise inference*. Threshold the LRT statistic images with a predetermined cluster‐forming threshold (*p*‐value *α* or statistic *u*_*α*_) and form clusters. Use a predefined grid of cluster size thresholds to define each cluster as detected or not.
4. Compute the family‐wise FPR and TPR:
*FPR*. Using the smoothed random noise images, for each threshold, the family‐wise FPR is the proportion of realizations having any (false) detections.
*TPR*. Using the heritability images, for each threshold, compute the proportion of true positive pixels (detected and *A* > 0) out of all possible (number of the *A* > 0 pixels). This is computed for each realization and averaged over realizations.
5. Plot the ROC curves and calculate the corresponding normalized AUC values.

#### 3.2.3 ROC‐based simulation results

**Figure 8:**
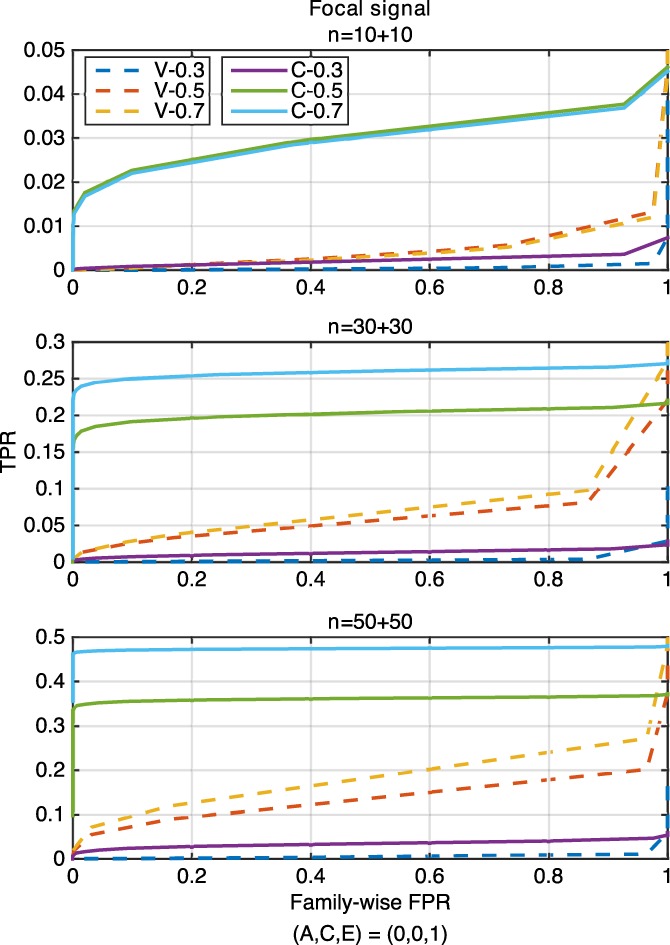
The ROC curve comparison of voxel‐ (“V,” dashed lines) and cluster‐wise (“C,” solid lines) inference approaches for three settings of (*A*, *C*, *E*) = (0.3, 0, 0.7), (0.5, 0.2, 0.3), (0.7, 0, 0.3), corresponding to *h* ^2^ = 0.3, 0.5, 0.7, for the focal signal with three sample sizes of 10 + 10 (upper), 30 + 30 (middle) and 50 + 50 (lower), where “V‐0.3” and “C‐0.3” represent the settings of voxel‐wise inference and *h* ^2^ = 0.3 and cluster‐wise inference and *h* ^2^ = 0.3, respectively [Color figure can be viewed at http://wileyonlinelibrary.com]

**Figure 9:**
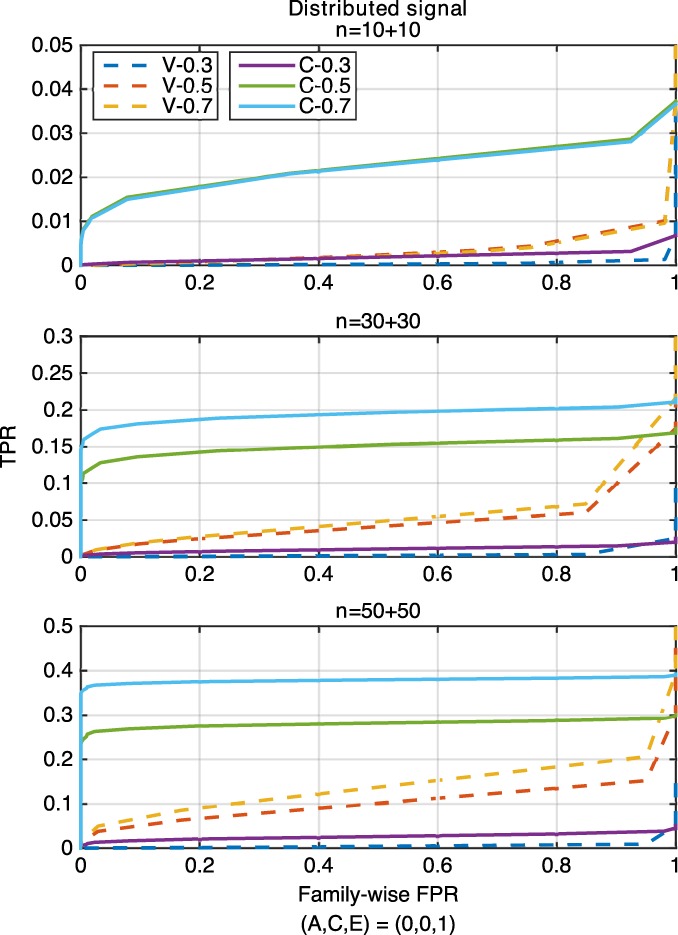
The ROC curve comparison of voxel‐ (“V,” dashed lines) and cluster‐wise (“C,” solid lines) inference approaches for 3 settings of (*A*, *C*, *E*) = (0.3, 0, 0.7), (0.5, 0.2, 0.3), (0.7, 0, 0.3), corresponding to *h* ^2^ = 0.3, 0.5, 0.7, for the distributed signal with three sample sizes of 10 + 10 (upper), 30 + 30 (middle) and 50 + 50 (lower), where “V‐0.3” and “C‐0.3” represent the settings of voxel‐wise inference and *h* ^2^ = 0.3 and cluster‐wise inference and *h* ^2^ = 0.3, respectively [Color figure can be viewed at http://wileyonlinelibrary.com]

**Figure 10:**
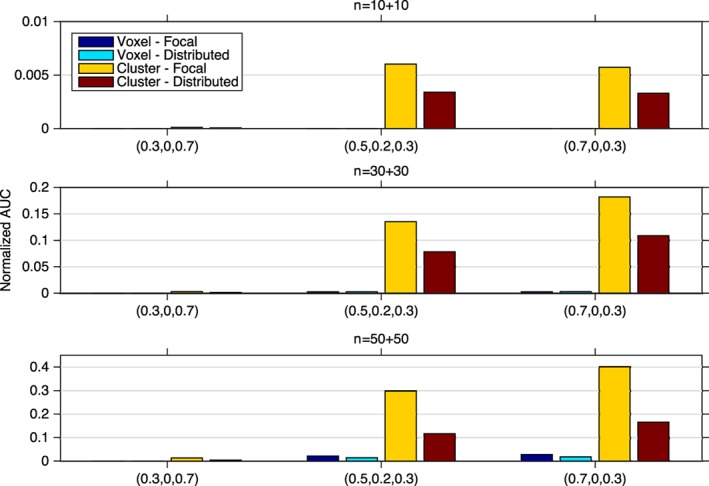
The normalized AUC (20 × AUC for FPR = 0:0.05) comparison of voxel‐ and cluster‐wise inference approaches for different (*A*, *C*, *E*) parameter settings, three samples of size *n* = 10 + 10, 30 + 30, 50 + 50, and two tested signals (focal and distributed) with positive heritability *h* ^2^ > 0 [Color figure can be viewed at http://wileyonlinelibrary.com]

As described above, a range of simulation settings are investigated for both voxel‐ and cluster‐wise inference approaches using APACE. For different extents of smoothness, the returned ROC curves have fairly similar shape, so we will only illustrate the ROC curves created by medium degree of smoothing with FWHM of three pixels, which are shown in Figures 8 and 9 for the simulated focal and distributed signals, respectively. The corresponding normalized AUC comparison is then shown in Figure 10.

For both focal and distributed signal, ROC curves of the cluster‐wise method are always above those of the voxel‐wise method, reflecting higher statistical power obtained for cluster‐ than voxel‐wise inference approaches. In general, for a particular family‐wise FPR level, the TPR value of both inference methods becomes larger when the sample size or the heritability is increased.

The normalized AUC values (Figure 10) show that the voxel‐wise method has poor performance overall for all simulation settings with negligible AUC values, while the cluster‐wise inference approach has much larger AUC values. While the absolute power is low here, reflecting the challenge of detecting nonzero heritability with just 100 subjects, these results show that the cluster‐wise approach is more sensitive to such spatial signals and demonstrates the value of such spatial statistics.

## 4 REAL DATA ANALYSIS

Here we report the heritability analysis of a working memory fMRI task. We illustrate the above‐mentioned heritability inference approaches including univariate LR‐SqD and permutations.

### 4.1 Real data acquisition

The experimental sample comprises *n* = 319 young and healthy participants from Queensland, Australia (199 females and 120 males), consisting of *n*_MZ_ = 150 MZ twins (75 pairs with 46 female and 29 male pairs), *n*_DZ_ = 132 DZ twins (66 pairs with 30 female, 11 male, and 25 opposite sex pairs) and *n*_S_ = 37 unpaired twins (22 female and 15 male). The age range of all these subjects is 20–28 years (mean ± *SD*: 23.6 ± 1.8). A 4T Bruker Medspec full‐body scanner was utilized and task‐related fMRI BOLD was acquired while participants performed a block design n‐back task, consisting of 0‐back and 2‐back conditions. Imaging preprocessing was implemented using SPM5 software in Matlab, including image realignment with a mean image generated, spatial normalization to the standard T1 template in MNI atlas space, spatial smoothing with an isotropic Gaussian kernel, removal of global signal effects, and the use of high‐pass and low‐pass filtering to discard uninterested signals. For each subject, the brain activation, measured as the 2‐back >0‐back *t*‐contrast images using a one‐sample *t*‐test, was extracted. Only areas of expected activation in the frontal and parietal regions are included in the mask, comprised of 14,627 voxels in total. Age, sex, and 2‐back performance accuracy (the percentage of correct responses) are included as the covariates in the statistical analysis (Blokland et al., 2011).

### 4.2 Real data results

The permutation‐based empirical distribution of maximum LRT statistic $T_{\pi }^{\max}$ gives 5% FWE threshold of $T_{\alpha }^{\mathrm{FWE}}=11.32$, and for the cluster‐wise results, the 5% FWE thresholds of maximum supra‐threshold cluster size $K_{\pi }^{\max}$ and maximum supra‐threshold cluster mass $M_{\pi }^{\max}$ are $K_{\alpha }^{\mathrm{FWE}}=62$ and $M_{\alpha }^{\mathrm{FWE}}=271.74$, respectively. The most significant FWE‐corrected *p*‐values are 0.007, 0.001, and 0.001 for voxel, cluster size and cluster mass statistics, respectively.

**Figure 11:**
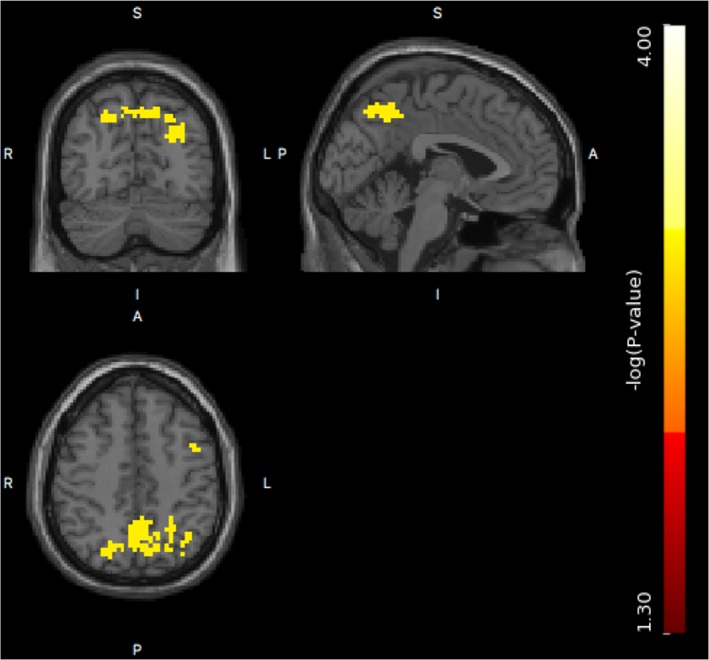
The log‐transformed FWE‐corrected *p*‐value image, that is, $-\log_{10}\left(p_{K}^{\mathrm{FWE}}\right)$, for supra‐threshold clusters with significant supra‐threshold cluster size [Color figure can be viewed at http://wileyonlinelibrary.com]

**Figure 12:**
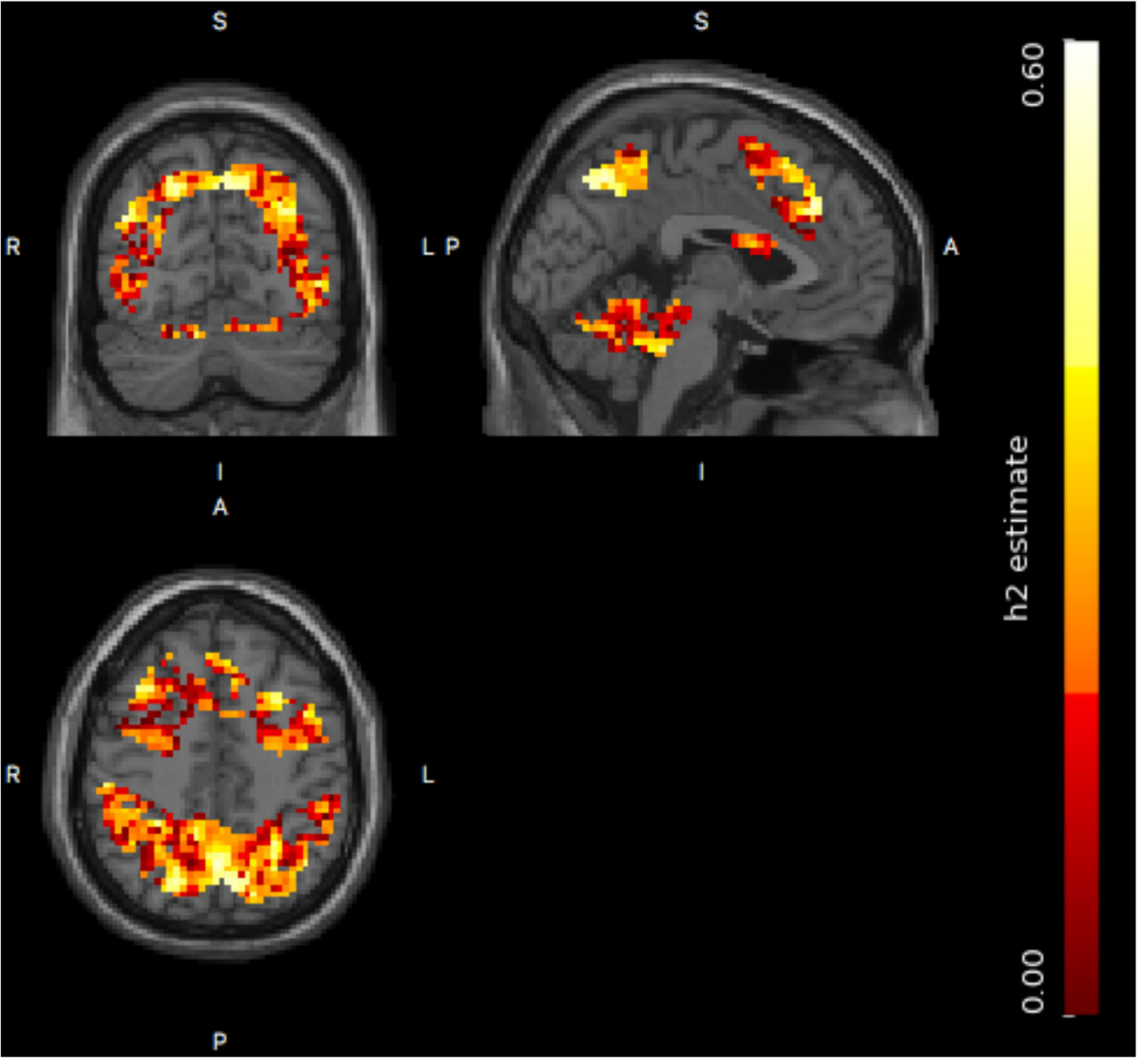
The heritability image for the masked brain regions. The heritability estimates vary between 0 and 0.59, and the heritability estimates of significant voxels using cluster size inference range from 0.18 to 0.59 [Color figure can be viewed at http://wileyonlinelibrary.com]

The supra‐threshold cluster tests found much larger significant brain regions than the single threshold test by comparing their FWE‐corrected *p*‐value images. For the voxel‐wise single threshold test, only two significant voxels were identified, while there were four clusters with a total of 634 voxels identified to be significant for the cluster‐wise tests. The FWE‐corrected *p*‐value image after log‐10 transformation, that is, −log_10_(*p*^FWE^), for significant supra‐threshold clusters with respect to size statistic is shown in Figure 11. The heritability estimate image is shown in Figure 12, where the heritability estimate ranges between 0 and 0.59, and the significant voxels based on cluster‐wise inference have a heritability range of 0.18 and 0.59. The most heritability‐significant regions found using both the single threshold test and the supra‐threshold cluster tests overlap with the most significant regions from the previous Mx analysis (Blokland et al., 2011).

## 5 CONCLUSION AND DISCUSSION

In this article, we have presented two novel linear regression‐based estimation methods for heritability inference in neuroimaging, trying to improve statistical power and reduce computational complexity. A simple LR‐SqD method based on linear regression modeling with squared differences of paired observations has been developed, and found to have comparable or even better estimation accuracy and statistical power relative to existing methods. LR‐SqD, as simple as Falconer's method, only requires linear regression to improve prediction accuracy. The univariate simulation study also showed that apart from Falconer's method, LR‐SqD is the most time‐efficient approach when compared with those likelihood‐based iterative methods, and will never encounter any convergence problems. The fast, accurate and noniterative properties of LR‐SqD make it more flexible and feasible to be applied for permutation inference.

A permutation‐based heritability inference approach by embedding LR‐SqD method in a permutation framework has also been developed. This permutation inference allows us to perform more exact heritability inference using LR‐SqD Perm at each voxel, to control the FWER, and also to consider alternative cluster‐wise imaging statistics. The fact that adjacent voxels or regions in a brain image tend to be structurally and functionally homologous can be exploited by spatial statistics like cluster size and mass. Our use of LR‐SqD, the fast and accurate noniterative method (free of any convergence issues), makes these spatially informed statistics more accessible. For equivalent FWERs, the cluster‐wise approach was found to have higher sensitivity, and thus more powerful in ROC‐based power simulations, which demonstrates the importance of such spatial statistics over voxel‐wise statistics and the need for permutation inference to take advantage of these cluster statistics. With few weak assumptions, permutation inference is a feasible alternative to the parametric approaches, which is even preferable in studies having small sample sizes or when the stronger assumptions of the parametric approaches cannot be met (Nichols & Holmes, 2001).

Except for LR‐SqD Perm, methods being compared in univariate simulations are asymptotic. We found our permutation‐based LR‐SqD method, LR‐SqD Perm, is more robust, being the most powerful approach for nearly all simulation settings. Other asymptotic LR‐SqD methods, LR‐SqD and LR‐SqD ReML, also have good power, and cluster inference methods have better detection power than voxel‐wise methods. A sample size of 1,000 is still insufficient, for some parameter settings, resulting in limited power (far below 80%) for detecting heritability, but at least we found all methods are valid with normally distributed errors except SOLAR, which is specially designed for family studies with large sample sizes of various degrees of relatedness. For Gaussian noise, although Falconer's method has poor estimation accuracy, it seems to work well with the power comparable to that of LR‐SqD Perm. However, it relies on the normality assumption to test for the equivalence of MZ and DZ correlations. For non‐Gaussian noise, the null distribution of LRT computed under the misspecified normality assumption can be inaccurate and the corresponding asymptotic null distribution of LRT based on Wilk's theorem is problematic, which results in inflated FPR as shown in our simulations. LR‐SqD Perm, which relaxes the assumption of normality, is the only applicable method that maintains valid FPR control in the case of non‐Gaussian error, and thus we suggest sticking to LR‐SqD Perm. During univariate evaluations, we found adding singletons can improve neither estimation accuracy nor statistical power. However, we still suggest including singletons in the statistical analysis since a better estimate of the phenotypic variance can be obtained with more data taken into account. Averaging across all the simulation settings, we found LR‐SqD is roughly 2.5 times faster than LR‐SqD ReML, and around 45.5, 84.8, and 995.7 times faster than SPM, OpenMx and SOLAR, respectively.

The LRT statistic for testing H_0_: *A* = 0 is not asymptotically pivotal and its distribution varies discontinuously across the parameter space depending on the true value of variance component *C*. The configurations of *C* on the parameter space can be partitioned into two cases: (1) *C* > 0, (2) *C* = 0. For standard Case (1), the reference distribution for the LRT involving one parameter on the boundary of the parameter space has been proven to be a half‐half mixture of $\chi_{0}^{2}$ and $\chi_{1}^{2}$ (Dominicus et al., 2006; Self & Liang, 1987). For nonstandard Case (2), under the null, both *A* and *C* are boundary parameters and the asymptotic distribution of the LRT statistic is a mixture of $\chi_{0}^{2},\;\chi_{1}^{2}$, and $\chi_{2}^{2}$ with mixing probabilities 1/2 − *p*, 1/2 and *p*, where 0 ≤ *p* ≤ 1/2 (Dominicus et al., 2006; Self & Liang, 1987). Even if *C* > 0 for Case (1), the asymptotic null distribution of the LRT statistic for a finite sample can be more similar to that for Case (2) when *C* is close enough to the boundary (Self & Liang, 1987). When the sample size tends to infinity or is sufficiently large, the asymptotic approximation is enhanced, and the tendency eases with the reference distribution more resembling that for Case (1). This leads to the conservativeness of the asymptotic LRT‐based tests when compared with the permutation‐based LR‐SqD Perm, and thus we recommend using the nonparametric permutation inference.

The existence of nonzero variance components *A* and *C* induces the familial correlation (Dominicus et al., 2006). When the true value of *A* is nonzero, testing the null hypothesis of no heritability is similar to testing for the familial correlation since it would be difficult to precisely separate the familial influences and explicitly distinguish between the *A* and *C* effects due to the inevitable noise, which has been revealed in univariate simulation evaluations. Therefore, increasing the variance parameter *C* may improve the power of the test for the null hypothesis of no heritability while holding the validity of the test. In addition, when *C* is zero, the test comparing the AE model against the E model could probably offer higher power than the test of ACE versus CE. However, the variance parameter *C* is unknown in reality and impulsively using the test of AE versus E would lead to inflated FPR and invalid conclusions with overestimated power.

We have developed a Matlab‐based tool “Accelerated Permutation Inference for the ACE Model (APACE)”, which provides different analysis approaches specialized for heritability inference based on LR‐SqD and is freely available at https://github.com/NISOx-BDI/APACE. Compared with the popular analysis tools such as OpenMx and SOLAR, APACE is designed specifically for neuroimaging data and is applicable for any sample sizes with controlled FPR. The use of the flexible permutation approach allows for any test statistics (e.g., LRT, cluster size, and so on) to be applied in computing the *p*‐values, and enabling parallel execution further accelerates the implementation. The current version of APACE can be adopted for the family design including twins, siblings and singletons, and the generalization of APACE for any family designs is possible with the use of the pedigree information.

## Data Availability

We have developed a Matlab‐based tool “Accelerated Permutation Inference for the ACE Model (APACE)”, which provides different analysis approaches specialized for heritability inference based on LR‐SqD and is freely available at https://github.com/NISOx-BDI/APACE.

## APPENDIX 1

### A PROOF

For convenience, the notation of **Y** can be simplified and written as **Y** = (Y_1_,  … , Y_*n*_)^′^ without loss of generality. We can write

$$\mathrm{SSD}=\left(n^{2}-n\right)s^{2}\left(Y\right),$$

where $\mathrm{SSD}=\sum_{l=1}^{\left(n^{2}-n\right)/2}D_{l}$ and $s^{2}\left(Y\right)=\sum_{i=1}^{n}{\left(Y_{i}-\bar{Y}\right)}^{2}/\left(n-1\right)$. To see this identity, we note that

Expanding this equation gives

$$\mathrm{SSD}=n\sum_{i=1}^{n}{\left(Y_{i}-\bar{Y}\right)}^{2}-\sum_{i=1}^{n}\sum_{j=1}^{n}\left(Y_{i}-\bar{Y}\right)\left(Y_{j}-\bar{Y}\right).$$

The second term is zero because

$$\begin{array}{l} \sum_{i=1}^{n}\sum_{j=1}^{n}\left(Y_{i}-\bar{Y}\right)\left(Y_{j}-\bar{Y}\right)=\sum_{i=1}^{n}\left\{\left(Y_{i}-\bar{Y}\right)\left[\sum_{j=1}^{n}\left(Y_{j}-\bar{Y}\right)\right]\right\}\\ =\sum_{i=1}^{n}\left[\left(Y_{i}-\bar{Y}\right)\left(n\bar{Y}-n\bar{Y}\right)\right]\\ =0 \end{array}$$

Thus, the proof is completed:

$$\mathrm{SSD}=n\sum_{i=1}^{n}{\left(Y_{i}-\bar{Y}\right)}^{2}=\left(n^{2}-n\right)s^{2}\left(Y\right).$$
